# Sex differences in the sustained attention of elementary school children

**DOI:** 10.1186/s40359-022-01007-z

**Published:** 2022-12-15

**Authors:** Barel Efrat, Tzischinsky Orna

**Affiliations:** Department of Behavioral Sciences and the Center for Psychobiological Research, The Max Stern Academic College of Emek Yezreel, Emek Yezreel, Israel

**Keywords:** Sleep, Actigraph, Children, Psychomotor vigilance test (PVT), Sex differences

## Abstract

**Background:**

The study investigates sex differences in sustained attention among children.

**Methods:**

Forty-five children (23 girls) from Grades 2–5 (mean age of 7.47 ± 0.73 years) wore an actigraph for a continuous five to seven days including school and non-school days. Sustained attention using the psychomotor vigilance test (PVT) was measured twice a day on two school days and on one non-school day.

**Results:**

No sex differences were found for sleep patterns. However, sex differences in PVT performance were documented. While boys were faster (shorter reaction time) and showed fewer lapses than girls, they showed higher number of false starts than girls, on both weekdays and weekends.

**Conclusions:**

The findings suggest that sex differences should been taken into account in studies investigating neurobehavioral functioning, particularly, sustained attention across various age groups.

## Introduction

Sustained attention, also called vigilant attention, refers to the attention control processes needed to preserve attention and task engagement over time [1]. It relates to a collection of cognitive processes which enable individuals to organize their behavior in response to current situational demands and future directions and is an essential part of executive function. Sustained attention is usually measured via computerized performance tasks requiring responses to target signals. The most widely used measure of sustained attention is the psychomotor vigilance test (PVT) due to its psychometric advantages over other cognitive measures [2]. Sustained attention has been shown to be influenced by the interaction between the circadian timing system and the homeostatic sleep regulatory system [3]. Studies have demonstrated the impact of sleep loss on PVT performance, which causes an overall slowing of response time, increasing lapses of attention, and modestly increasing false starts (e.g., [4]). Apart from its sensitivity to circadian and homeostatic modulation, sustained attention is also susceptible to the effects of age [5] and sex [5, 6].

Sex differences in sustained attention have been studied across various age groups. Venker et al. [7] investigated sex differences in the mean and median PVT reaction time (RT) of children aged 6–11. They showed that boys were faster than girls; however, these differences tended to disappear with age. Beijamini et al. [6] evaluated the influence of gender on the PVT performance of adolescents. They found that boys were faster and had fewer lapses than girls. Blatter et al. [5] examined sex differences in the PVT performance of young adults (aged 20–31) and older adults (aged 57–74). They found that men were faster than women, but women were more likely to avoid false starts. They suggested that these patterns of sex differences may imply the use of different strategies to achieve optimal results: women aim to avoid errors, while men aim to be as fast as possible.

Such a suggestion begs the question of whether sex differences in achievement strategies are apparent in early stages of development, and the present study therefore chose to investigate sex differences in the sustained attention of children. Previous studies have found differences between boys and male adolescents and girls and female adolescents, showing the former to present shorter reaction times, lower error rates, and higher risk-taking tendencies (e.g., [7–9]). Similar to the premise regarding sex differences in achievement strategies among adults [5], it has been suggested that sex differences in performance among children correspond to sex differences in response styles [8]. It has been suggested that girls tend to adopt a more cautious approach to selecting answers than boys which, in a limited time frame, would lead to less accurate response patterns. Studies have provided support for this assertion by showing that girls display more conscientiousness [10] and cautiousness [11, 12] than boys. Further support has come from neuroimaging studies. For example, Cornblath et al. [13] investigated the neurobiological basis of sex differences in executive functions, specifically regarding impulsivity among children. They measured sex differences in network controllability and impulse control using the false positive rate in the continuous performance test (CPT) and found that local sex differences in controllability predict false positive responses. Furthermore, superior parietal modal controllability was higher in females and found negatively associated with impulsivity, leading the authors to suggest that these regions are involved in impulse control in females.

It is important to note, however, that not all studies showed consistent findings. Some found no sex differences (children aged 6–14) (e.g., [14]), and one study demonstrated that girls had greater attentional capacity than boys (aged 3–7) [15]. This inconsistency may be due to the different stimuli used. The most frequent test used is the PVT, followed by the CPT and its many versions. It has been suggested that sustained attention may constitute multiple distinct constructs and the PVT and the CPT are not pure measures of sustained attention [16]. Furthermore, the CPT requires longer duration and most of the assessment versions were used among adults [17].

Numerous studies have shown that sustained attention is highly influenced by sleep quality. This is, to the best of our knowledge, the first study to examine sex differences in the PVT performance of children by controlling for sleep quality via objective measurements (actigraphy). In the present home-based study, sex differences in PVT performance were evaluated in a natural setting via multiple observations (several weeknights and weekend nights) of 45 children. Only a few studies have addressed sex differences in the sustained attention of children (e.g., [7, 18]); they did not, however, control for sleep quality. Given that sustained attention is highly sensitive to sleep loss, the present study controlled for this variable through multiple observations of sleep via actigraphic recording.

Previous studies explored the relationship between sleep quality and cognitive performance among children. For example, Astill et al. [19] conducted a meta-analysis and found a modest association between sleep duration and cognition among children. They reported that even sustained attention and memory, both of which are sensitive to poor sleep, showed no significant association with sleep duration. A possible explanation of these results is that brain immaturity may prevent children from feeling poor sleep and fatigue. Recently, Short et al. [20] showed in a meta-analytic review that only a few studies have examined the association between the sleep duration and optimal cognitive performance of school-age children. Both of these meta-analyses demonstrated that sleep duration is associated with specific cognitive function but not with others and that sleep duration was not associated with sustained attention. Sleep duration may vary between weekend and weekdays nights; however, given that it was not found associated with attention, we assumed that the role of sex differences in sustained attention will not differ between weekend and weekday nights. The present study therefore looked to measure sleep patterns but did not expect sex differences in sustained attention to depend on sleep.

Regarding sex differences in sleep patterns and sleep-related difficulties, data from several studies suggested that among adolescent, girls tend to report sleep difficulties more frequently than boys [21–24]. In contrast to adolescents, the results among children are inconsistent. Whereas some studies observed different sleep patterns between boys and girls with more sleep difficulties among boys than girls [25], others found no gender differences [24] or more frequent difficulties among girls than boys [26]. Recently, Lewien et al. [27] indicated that sleep-related difficulties were more frequent among boys in the child sample and among girls in the adolescent sample.

The present study therefore hypothesized that: 1. there are no sex differences in sleep patterns; and 2. there are sex differences in PVT performance, with boys being faster (shorter RT) and showing fewer lapses than girls but showing a higher number of false starts than girls both on weekdays and at weekends.

## Methods

### Participants

Study participants included 45 children (23 girls; mean age 7.43 ± 0.90 years; 22 boys; mean age 7.50 ± 0.51 years) from normative elementary schools, Grades 2–5 (most in Grades 3 and 4), in urban and rural middle-class communities in northern Israel (mean age 7.47 ± 0.73 years). All participants began school between 7:45 and 8:50 in the morning, 42.4% had 2 days off a week (Friday and Saturday), and 57.8% had only one day off (Saturday). Of all participants, 82.2% did sport between one and seven times a week and 8.9% reported drinking coffee during the day. While 15.6% were diagnosed with ADHD (6 boys, 1 girl), no differences were found between children with/without ADHD in sleep or cognitive measures, and they were therefore taken as one group.

### Materials

#### Cognitive performance

Participants completed a visual PVT twice a day: in the morning after wake-up and before bedtime. We examined their cognitive functioning in the morning and the evening because sleepiness is affected by the combination of circadian phase and time awake, as was quantified in laboratory sleep deprivation studies [28]. In field studies the daily time course of sleepiness was found to be U-shaped: moderate levels upon awakening which decrease and reach a minimum in the early evening and then increase in the evening hours (before bedtime) to levels that were higher than those upon awakening [29]. The PVT task is sensitive to sleep loss and circadian phase [30, 31] and has been employed for the last 30 years as a sensitive test of sustained attention [32]. This simple measure of reaction time (RT) to repetitive stimuli has become recognized as a highly sensitive and effective tool for measuring degradation of sustained attention performance under sleep deprivation or partial sleep deprivation or changes in circadian phases [30, 31].

The PVT-B (Joggle Research, Seattle, WA) is a three-minute long sustained attention RT task, which, in the present study, was performed on an iPad. It is a validated measure of sustained attention with high test–retest reliability and low learning effects [1]. Participants were instructed to maintain vigilant attention on a target box and to respond as quickly as possible to the appearance of a stimulus while avoiding premature responses. The outcome measures of the present study were mean RT, lapses, and false starts. Participants were requested to press on the screen to stop the counter, responding as quickly as possible but avoiding pressing on the screen when the counter was not displayed (i.e., false starts). The interstimulus interval, defined as the period between the last response and the appearance of the next stimulus, varied randomly from 2 to 10 s [33].

#### Sleep measures

Objective sleep patterns were measured using an actigraph (Mini-Act, AMA-32, AMI)—a small movement sensor that continuously records body mobility, accumulates movement over one minute, and causes the sensor signal to cross a fixed reference signal. Sleep/wake measures were estimated from actigraphy data using a validated algorithm [34].

Participants wore the actigraph on the wrist of their non-dominant hand for one week including weekdays (four–five nights) and weekends (one night before non-school day) in a natural environment (home). Actigraph recordings were scored using the event marker button for subjective sleep and wake times. The analysis of the actigraph recoding (using the program Action) provided an estimation of participants’ sleep onset times and wake-up times, sleep latency, time in bed (from sleep onset to wake-up time), true sleep minutes (only minutes of sleep), wake after sleep onset (WASO), and sleep efficiency.

### Procedure

Participants were collected using the snowball method. The research assistant met with potential participants and their parents at home. The parents of those children who agreed to participate in the study signed an informed consent. Participants wore the actigraph continuously for five to seven days, on both school and non-school days, and completed the PVT for three minutes. Participants were required to maintain a regular sleep pattern on school and non-school days.

The ethics committee at Emek Yezreel College (no: EMEK YVC 2018-48) approved this study.

### Statistical analysis

Scores were averaged per individual on weekdays. PVT measures were not normally distributed and were therefore subject to a log 10 transformation that normalized their distribution. Independent sample *t*-tests were calculated for sex differences in actigraph sleep patterns between weekday and weekend measures. Bivariate correlations were conducted between sleep duration (calculated as time in bed multiplied by sleep efficiency) and PVT measures. Bonferroni corrections for multiple comparisons were calculated. PVT performance tests were analyzed via repeated-measures analysis of variance (sex × day × time) with age, ADHD, and mean sleep duration as covariates. For all the ANOVA tests, whenever Mauchly’s test indicated a violation of sphericity assumption, Greenhouse–Geisser corrections were used. Effect-size analyses for t-tests were calculated based on Cohen’s *d*, whereas effect-size for ANOVA were calculated using partial eta-squared (*η*^2^_*p*_).

## Results

Sleep patterns as measured through actigraph recordings are presented in Table 1. A series of two-way repeated-measures ANOVAs with sex (boys, girls) and day (WD, WE) as the independent variables was conducted. A significant sex × day interaction was found for sleep duration. Further analyses revealed no significant effect of sex on weekdays [*t* (35) = 1.89, *p* > 0.05; Cohen’s *d* = 0.62] or weekends [*t* (40) = 1.84, *p* > 0.05; Cohen’s *d* = 0.57]. No other significant interactions were found. No significant main effect for either sex or day was found for sleep patterns (Table 1).

**Table 1:** Means (SD), F, and *η*^2^_*p*_ for actigraph sleep measures, boys vs. girls, weekdays and weekends (N = 45)

	Weekdays (WD)		Weekend (WE)		F	*η*^2^_*p*_
	Boys	Girls	Boys	Girls		
Bedtime (hh:mm)	21:19 (0:19)	21:38 (0:32)	23:04 (1:04)	22:37 (1:37)	2.13	0.06
Wake-up time (hh:mm)	6:53 (0:31)	6:49 (0:25)	7:58 (1:09)	8:18 (1:12)	1.32	0.04
Time in bed	575.18 (34.33)	551.07 (49.42)	535.70 (66.25)	581.21 (80.62)	6.49*	0.16
WASO	27.41 (15.87)	24.98 (19.65)	24.58 (14.16)	38.18 (28.58)	3.56	0.10
Sleep latency	19.56 (14.94)	17.24 (9.56)	15.23 (10.01)	14.38 (12.96)	0.00	0.00
Sleep efficiency	95.31 (3.00)	95.44 (3.53)	95.24 (2.68)	93.27 (5.13)	2.12	0.06

*WASO* minutes of wake after sleep onset

*p < 0.05

The correlation did not remain significant after performing Bonferroni corrections for multiple comparisons

We also checked for associations between sleep duration and PVT measures. Bivariate correlations did not demonstrate significant results (see Table 2).

**Table 2:** Correlations between PVT measures and sleep duration in morning and evening, weekdays and weekends (N = 45)

	Weekdays (WD)		Weekend (WE)	
	Morning	Evening	Morning	Evening
Mean RT_log10_	0.08	0.23	− 0.02	− 0.03
Lapses_log10_	0.04	− 0.01	− 0.15	0.14
False starts	− 0.26	0.14	0.43*	0.09

*p < 0.05

The correlation did not remain significant after performing Bonferroni corrections for multiple comparisons

For PVT measures (mean RT, lapses, false starts), a series of three-way repeated-measures ANOVAs with sex (boys, girls), day (WD, WE), and time (M, E) as the independent variables was conducted, with age, ADHD, and mean sleep duration as covariates. A significant effect of sex was found [*F* (1, 25) = 4.34, *p* < 0.05; *η*^2^_*p*_ = 0.16] for mean RT, with boys demonstrating shorter mean RTs than girls (see Table 3). A significant sex × time interaction [*F* (1, 25) = 8.17, *p* < 0.01; *η*^2^_*p*_ = 0.28] was also found. Further analyses revealed a significant effect of sex for morning [*t* (42) = 1.70, *p* < 0.05 1-tailed; Cohen’s *d* = 0.48] with boys (M = 2.57, SD = 0.13) being faster than girls (M = 2.64, SD = 0.16); no significant difference was found for evening [*t* (43) = 1.53, *p* > 0.05; Cohen’s *d* = 0.71]. There was no significant sex × day interaction [*F* (1, 25) = 0.13, *p* > 0.05; *η*^2^_*p*_ = 0.01], day × time interaction [*F* (1, 25) = 0.48, *p* > 0.05; *η*^2^_*p*_ = 0.02], or sex × day × time [*F* (1, 25) = 2.04, *p* > 0.05; *η*^2^_*p*_ = 0.08]. There was no significant main effect for day [*F* (1, 25) = 0.33, *p* > 0.05; *η*^2^_*p*_ = 0.01] or time [*F* (1, 25) = 0.00, *p* > 0.05; *η*^2^_*p*_ = 0.00].

**Table 3:** Means (SE) for PVT, weekdays vs. weekend (N = 42)

	Weekdays (WD)		Weekend (WE)	
	Boys	Girls	Boys	Girls
Mean RT_log10_	2.55 (0.02)	2.57 (0.02)	2.51 (0.04)	2.65 (0.04)
Lapses_log10_	0.91 (0.07)	1.01 (0.07)	0.86 (0.06)	1.14 (0.06)
False starts	9.36 (1.14)	8.07 (1.14)	13.77 (1.95)	8.13 (1.95)

WD = weekdays; WE = weekend; Mean RT_log10_ = log transformed PVT mean RT; Lapses_log10_ = log transformed PVT number of lapses

For PVT lapses, a significant effect of sex was found [*F* (1, 25) = 5.04, *p* < 0.05; *η*^2^_*p*_ = 0.18] with boys having fewer lapses than girls. A significant sex × day interaction [*F* (1, 25) = 4.48, *p* < 0.05; *η*^2^_*p*_ = 0.16] was also found. Further analysis revealed that sex differences in PVT lapses were found for weekends [*F* (1, 35) = 13.77, *p* < 0.01; *η*^2^_*p*_ = 0.28] with boys having fewer lapses than girls. However, for weekdays no significant differences were found [*F* (1, 37) = 0.80, *p* > 0.05; *η*^2^_*p*_ = 0.02]. There was no significant sex × time interaction [*F* (1, 25) = 1.10, *p* > 0.05; *η*^2^_*p*_ = 0.05], day × time interaction [*F* (1, 25) = 0.00, *p* > 0.05; *η*^2^_*p*_ = 0.00], or sex × day × time [*F* (1, 25) = 3.25, *p* > 0.05; *η*^2^_*p*_ = 0.12]. There was no significant main effect for day [*F* (1, 25) = 0.59, *p* > 0.05; *η*^2^_*p*_ = 0.03] or time [*F* (1, 25) = 0.42, *p* > 0.05; *η*^2^_*p*_ = 0.02].

For PVT false starts, a significant effect of sex was found [*F* (1, 25) = 3.31, *p* < 0.05 1-tailed; *η*^2^_*p*_ = 0.13]. The average level of false starts was lower among girls than boys. There was no significant sex × day interaction [*F* (1, 25) = 2.56, *p* > 0.05; *η*^2^_*p*_ = 0.03], sex × time interaction [*F* (1, 25) = 2.07, *p* > 0.05; *η*^2^_*p*_ = 0.08], day × time interaction [*F* (1, 25) = 0.01, *p* > 0.05; *η*^2^_*p*_ = 0.00], or sex × day × time [*F* (1, 25) = 3.30, *p* > 0.05; *η*^2^_*p*_ = 0.13]. There was no significant main effect for day [*F* (1, 25) = 0.00, *p* > 0.05; *η*^2^_*p*_ = 0.00] or time [*F* (1, 25) = 0.18, *p* > 0.05; *η*^2^_*p*_ = 0.01].

## Discussion

Based on earlier observations regarding sex differences in sustained attention across various age groups, we investigated performance differences among children. Furthermore, given that sustained attention is sensitive to sleep loss, we objectively evaluated sleep quality and time in bed using actigraphic objective measurements. The time in bed of weekdays and weekend reflected the recommended time in bed (more than 9.5 h) [35].

Our findings supported the first hypothesis by showing no sex differences in sleep patterns. These findings are in line with previous reports suggesting no sex differences before puberty. For example, Jenni and LeBourgeois [36] reported no sex differences in sleep EEG power bands in prepubescent boys and girls, and Feinberg et al. [37] reported no sex differences in NREM delta power between ages 9 and 11. The authors suggested that the emergence of sex differences at puberty are due to sexual maturation. Furthermore, the authors also suggested that sex hormone regulatory mechanisms may play a role in the decline of NREM delta power density.

Next, we examined the association between sleep duration and PVT performance. In accordance with previous meta-analyses [19, 20], we did not find any correlation. While among adults and adolescents sleep loss results in inattention, among children domains typically sensitive to sleep loss, such as sustained attention, show no significant relationship with sleep duration. Astill et al. [19] suggested that sustained attention requires activation of the frontoparietal networks that are compromised following sleep loss among adolescents and adults, whereas in children this network is still quite immature.

Our second hypothesis regarding sex differences in PVT performance was also confirmed. Under sleep satiation, boys presented shorter PVT RT and fewer lapses than girls, while girls showed fewer false starts than boys. This is in accordance with previous studies which demonstrated that boys are faster and have fewer lapses on sustained attention tasks. For example, Lin et al. [18] examined performance on the continuous performance test (CPT) of children aged 6–15, while Venker et al. [7] examined performance on the PVT of children aged 6–11. Both studies documented sex differences in sustained attention in favor of boys. However, not all studies yielded this pattern of differences between boys and girls (e.g. [14, 15]). These two studies, for example, used different versions of the CPT. Their inconsistency may be due to differences in the measures of each stimulus used; for example, the nature of commission errors and time length of administration for the PVT are different from the CPT [38]. Recently, Rouse et al. [39] used both PVT and CPT to evaluate the effects of medication on attention and other aspects of cognitive functioning among children diagnosed with excessive daytime sleepiness. They found that specific measures of each test indicated other aspects of improved attention in the medicated group. Specifically, the PVT false starts indicated an increase in attention, vigilance, and alertness, and CPT omissions indicated a decrease in the failure to respond to stimuli while children were medicated. These findings call for further research directly comparing PVT and CPT among children to deepen our understanding of the multiple aspects of sustained attention.

We further found that sex difference in PVT RT was moderated by the time of day. Sex differences favoring boys were apparent at morning, while no sex difference was found in the evening. We turned to literature on individual differences in chronotype to shed light on these results. A meta-analysis suggested a significant effect of sex on morningness, with men tending to score significantly more toward eveningness than women [40]. Other studies focused on the role of chronotype on cognitive abilities. For example, Preckel et al. [41] reported a positive association between eveningness and cognitive ability, while Roberts and Kyllonen [42] found that higher scores on eveningness were associated with higher scores on a working memory task, even though the task was performed in the morning. These results suggest that there may be an interaction between sex and chronotype on cognitive performance. To address this Escribano and Díaz-Morales [43] examined the role of sex and chronotype on sustained attention among adolescents. They found that boys presented higher attention levels than girls and that evening type boys tended to perform better than morning type boys and evening type girls even though they were tested in the morning. While the present study did not include chronotype, it should, in our opinion, be considered in future studies. The current preliminary results among children call for replication in future studies including variables such as chronotype, sex, age, and time of day and their interaction exploring their role in attention performance.

We also detected sex differences in the number of PVT false starts, with girls having less false starts than boys. This finding is consistent with previous studies among adults, showing that women commit fewer false starts in vigilance performance than men (e.g., [44]). Blatter et al. [5] explored sex and age differences in the PVT and showed that women tended to inhibit their PVT response to maintain accuracy while men demonstrated faster RT than women. The authors suggested that women and men may use different strategies to achieve optimal results. This is in line with sex differences found in other cognitive domains. For example, Adam et al. [45] demonstrated sex differences in choice RT. Participants performed RT tasks that required a verbal response to a spatial location target stimulus. Men and women showed different RT patterns as a function of stimulus location, and the authors suggested that this may reflect differences in processing strategy. Support for this assertion comes from neuroimaging studies that showed increased activation in temporo-occipital, posterior cingulate, and cerebellar cortices in females in comparison to males during cognitive control and sustained attention contrast tasks [46].

As expected, our results showed sex differences in PVT measures for weekend and weekend nights. While previous meta-analyses [19, 20] did not find an association between sleep duration and sustained attention among children, they did document an association with other cognitive domains (e.g., executive function). We therefore hypothesized that a sex difference effect would be found for both weekend and weekday nights regardless of sleep duration. Investigating differences in sleep patterns in the present sample revealed no significant effect; in other words, our participants had sufficient sleep duration [35] on both weekend and weekday nights. As a result, we had no further opportunity to explore the role of sleep loss in the association between sex differences and sustained attention. Future studies should pursue this objective by including various populations with a range of sleep duration.

Despite its important findings, certain limitations of this study should be noted. First, despite the fact that studies investigating characteristics that may vary significantly within populations often require larger sample sizes, the current study has a small sample size. Interpretations should thus be made with caution. Second, regarding sample characteristics, the children participating in the present study were healthy and were good sleepers. The extent to which our findings can be generalized to other populations is therefore unknown. Third, the children were recruited from middle-class communities with sleep regimes that supported sufficient time in bed on both weekend and weekday nights. Parental supervision has been found essential for the maintenance of healthy sleep habits over time [47]. Future studies should examine other populations to assess the role of sleep deprivation on sex differences in sustained attention. Lastly, the present study did not include an examination of personality traits. A few studies have found a correlation between sustained attention and personality traits, such as the Big Five conscientiousness [48]. Furthermore, previous studies exploring sex differences in cognitive performance and controlling for personality variables have documented the mediating role of personality traits in the relationship between sex differences and cognitive performance (e.g., [49]). However, there is, to the best of our knowledge, no previous report on the role of personality traits in the association between sex differences and sustained attention. We recommend future studies to explore the mechanisms underlying sex differences in sustained attention.

## Conclusions

This is, to our knowledge, the first home-based study (field study) to document sex differences in the PVT performance of children using objective measurements to control for sleep quality. Our study supports previous suggestions regarding differential cognitive strategies for girls and boys. Future studies might deepen our understanding of the differential strategies used by females and males across developmental landmarks by also using neuroimaging to unravel sex differences in activation patterns throughout development. An understanding of the mechanisms underlying sex differences in sustained attention will provide insight into sex-related differences in other functioning domains, such as recreational and occupational preferences and functioning. Furthermore, a growing body of research on sex differences in cognitive performance and sustained attention in particular, unraveling the similarities and differences between females and males, will enable the development of differential training programs and evaluation methods for girls and boys, with an emphasis on the strengths and weaknesses of both sexes based on the principles of brain plasticity.

## Data Availability

The datasets used during the present study are available from the corresponding author on reasonable request.

## Abbreviations

PVT: Psychomotor vigilance test
RT: Reaction time
CPT: Continuous performance test
ADHD: Attention deficit/hyperactivity disorder
WASO: Wake after sleep onset
EEG: Electroencephalogram
NREM: Non rapid eye movement
